# When AI advice agrees or disagrees with human judgment: trust, self-efficacy, psychological resilience, and adaptive responses in physical education

**DOI:** 10.3389/fpsyg.2026.1972538

**Published:** 2026-10-21

**Authors:** Haoqun Yang

**Affiliations:** 1 School of Interdisciplinary Studies, Lingnan University, Tuen Mun, Hong Kong SAR, China; 2 NTU Entrepreneurship Academy (NTUpreneur), Nanyang Technological University, Singapore, Singapore

**Keywords:** human-AI collaboration, judge-advisor system, machine trust, physical education, psychological resilience, self-efficacy

## Abstract

As AI-based decision-support systems become increasingly embedded in physical education and coaching practice, agreement and disagreement between algorithmic advice and participants’ immediate scenario judgments have become important but theoretically underexamined features of human-AI collaboration. Grounded in a shared Judge-Advisor System paradigm, four scenario-based experiments (total *N* = 859) manipulated whether an experimentally presented AI recommendation was consistent or conflicting with each participant’s prior judgment and tested machine trust and collaborative decision-making self-efficacy as psychological pathways to self-reported collaborative adaptive responses. Psychological resilience and rationale provision were examined as study-specific boundary conditions. Conflict significantly reduced machine trust (Study 1, *d* = 1.061) and self-efficacy (Study 2, *B* = −0.687), and both measured mediators carried significant negative indirect associations with adaptive responses (Study 1, *B* = −0.428; Study 2, via trust *B* = −0.201, via self-efficacy *B* = −0.127). Psychological resilience attenuated the between-condition difference in self-efficacy in Study 3 (index of moderated mediation = 0.115). In Study 4, rationale provision moderated the condition-to-trust association, *F*(1, 239) = 11.348, *p* < 0.001, partial η^2^ = 0.045: the conflict effect was −0.811 in the black-box condition but −0.182 when a rationale was provided. The manipulation therefore concerns advice-judgment agreement rather than a direct experimental comparison of data-driven judgment with established experiential intuition. Because the scenarios did not establish objective recommendation accuracy, the moderation patterns are interpreted as changes in psychological response rather than as evidence that preserving trust or self-efficacy necessarily improves calibration, decision quality, or welfare.

## 1 Introduction

Over the past several years, as artificial intelligence has steadily made its way into physical education and athletic training practice, data-driven analytic systems have come to be embedded across fitness monitoring, injury-risk alerting, and tactical-optimization advising, gradually assuming a role as a routine decision-support tool for teachers and coaches (Zhou et al., 2024; Wang and Wang, 2024; He et al., 2024; Tan et al., 2025). What has largely driven this adoption is the capacity of such systems to process large-scale, multidimensional data that experiential judgment alone struggles to encompass, surfacing patterns not readily apparent to intuition. Worth bearing in mind, however, is that these systems remain, at their core, probabilistic and pattern-recognition-based in nature, and a fine-grained understanding of specific contexts, individual differences, or the relational texture of teaching is something they seldom achieve. It is precisely here, once such systems enter a domain as deeply reliant on embodied experience and in-the-moment judgment as physical education, that their underlying limitations tend to come into sharper relief. The empirical contrast examined in the present studies is narrower than a data-versus-experience comparison: the manipulation varies whether an AI recommendation agrees or disagrees with the participant’s immediately stated judgment in a specific scenario.

From this arises a more specific question: when an AI recommendation diverges from a participant’s immediate judgment in a concrete physical-education scenario, how does that disagreement shape subsequent collaboration? Such disagreement is not necessarily rare or peripheral; it can arise whenever human and algorithmic assessments rely on different information or weighting of evidence (Vaccaro et al., 2024). A system might, for instance, flag elevated injury risk for a given student, whereas the teacher, drawing on an embodied familiarity with that student’s physical condition and training history, may judge the risk to be minimal. Alternatively, an algorithm might recommend a particular adjustment to training load, only for the coach’s own in-the-moment observation to suggest otherwise. Neither clear decision-making guidance nor a systematic account of teachers’ psychological responses has, as things stand, been developed for this kind of advice-judgment disagreement, leaving it as a practical problem within human-AI collaborative decision-making research still very much in need of clarification.

Turning to existing scholarship, several lines of inquiry have, each in their own way, engaged with questions of trust and decision-making in human-AI collaboration. From research on automation trust have emerged the seemingly contradictory phenomena of algorithm aversion and algorithm appreciation, suggesting that trust formation may depend considerably on situational cues (Afroogh et al., 2024; Holbrook et al., 2024). From a capability-belief perspective, self-efficacy research has offered some account of the psychological fluctuations decision-makers experience when confronting complex tasks, and individual variation in coping with stressful situations has, in turn, been addressed to some extent within the resilience literature (Bergdahl and Sjöberg, 2025; Chiu et al., 2025). What deserves note, though, is that these lines of research have largely developed along separate tracks, rarely brought together within a single integrative framework. The possibility that trust, efficacy, and resilience jointly operate, whether as parallel mediators or subject to particular moderating boundary conditions, has yet to receive adequate empirical testing.

At the same time, a review of where human-AI collaborative decision-making research has thus far concentrated reveals a marked emphasis on domains oriented toward objective quantification, medical diagnosis, financial forecasting, and judicial sentencing among them, while physical education, a setting in which judgment often incorporates embodied, situational, and relational information alongside formal data, has received comparatively little attention. Whether existing theoretical frameworks can be meaningfully extended to this context remains, for the most part, an open empirical question.

More important still, prior research has documented reductions in trust and efficacy under disagreement, but such reductions are not necessarily maladaptive. A decrease in trust may reflect rational calibration as well as disagreement-based resistance; without holding system accuracy and evidence quality constant, it is not interpreted here as algorithm aversion (Kostick-Quenet et al., 2024). The present research therefore tests whether rationale provision attenuates the trust response to disagreement and whether psychological resilience buffers between-condition differences in self-efficacy, without assuming that every reduction in trust represents collaboration failure.

In light of these considerations, the present research examines whether machine trust and collaborative decision-making self-efficacy constitute simultaneous indirect pathways linking the experimental consistency condition to self-reported collaborative adaptive responses. It further examines psychological resilience and rationale provision as study-specific boundary conditions for the self-efficacy and trust pathways, respectively. Because the mediators were measured rather than experimentally manipulated, the indirect effects are interpreted as evidence consistent with mediation rather than definitive proof of causal psychological mechanisms.

Toward this end, four progressively structured, scenario-based experiments were conducted within a shared Judge-Advisor System paradigm. The studies provide accumulating, non-pooled evidence: Study 1 tests the trust pathway; Study 2 adds the simultaneous self-efficacy pathway in a different scenario; Study 3 tests resilience as a first-stage moderator of the self-efficacy pathway; and Study 4 tests rationale provision as a moderator of the trust pathway. Because the two moderators were examined in different study datasets, their findings are synthesized conceptually and are not interpreted as a jointly estimated dual-moderation model.

The study contributes at two levels. Theoretically, it integrates trust, self-efficacy, and resilience within a cross-study conditional-process account of human-AI collaborative decision-making in physical education while preserving the distinction between study-specific estimates. Practically, the findings identify candidate boundary conditions for psychological responses to disagreement, but they do not validate an interface intervention or establish that attenuating reductions in trust or self-efficacy is beneficial. Because the studies use hypothetical scenarios, manipulated recommendations without objectively established accuracy, and predominantly self-reported outcomes, any implication for AI-interface design or real-world implementation should be treated cautiously and as hypothesis-generating rather than prescriptive (Schoeffer et al., 2024; Cecil et al., 2024).

## 2 Literature review and theoretical framework

### 2.1 Human-AI collaborative decision-making and the JAS paradigm

Research on human-AI collaborative decision-making has evolved from work on automation trust toward behavioral decision-making approaches that examine how people revise judgments after receiving advice (Glickman and Sharot, 2025). The Judge-Advisor System (JAS) paradigm formalizes this process by separating an initial judgment, an advisory input, and a final judgment, allowing researchers to quantify advice taking and judgment revision (Harvey and Fischer, 1997; Bonaccio and Dalal, 2006). The paradigm has subsequently been applied to human-AI settings to study the adoption, discounting, and resistance of algorithmic advice, including questions of appropriate reliance and trust calibration (Vaccaro et al., 2024; Tatasciore and Loft, 2025; Visser et al., 2025). Weight of Advice (WOA) is a commonly used advice-taking index in this tradition, but its interpretability depends on a nonzero numerical difference between the initial judgment and the advice; accordingly, the present study treats WOA cautiously and does not equate it with decision quality. Human-AI research has concentrated heavily on analytically structured domains, whereas physical education requires embodied, situational, and relational judgment that is less well represented in the existing evidence base (Wang and Wang, 2024; Zhou et al., 2024). This contextual difference motivates testing whether trust and efficacy-related processes observed elsewhere also appear in physical-education decision scenarios.

### 2.2 Theoretical foundations of algorithmic trust

Trust is widely regarded as a core construct in human-automation and human-AI collaboration, reflecting judgments about system competence, reliability, and dependability that can shape reliance behavior (Jian et al., 2000; Afroogh et al., 2024). Contemporary work also distinguishes trust from appropriate reliance: high trust is not necessarily beneficial when advice is inaccurate, and reduced trust is not necessarily maladaptive when skepticism is warranted (Kostick-Quenet et al., 2024; Visser et al., 2025). Findings labeled algorithm aversion and algorithm appreciation further indicate that reactions to algorithmic advice vary with task characteristics, prior experience, and information about system performance (Afroogh et al., 2024; Holbrook et al., 2024; Kahr et al., 2024; Nicolson et al., 2025). In the present model, agreement versus disagreement between algorithmic advice and the participant’s initial judgment is therefore treated as the manipulated situational predictor, not as a moderator. A trust reduction following disagreement may represent rational calibration, confirmation-based resistance, or both; because the scenarios do not establish objective ground truth, the present studies do not equate lower trust with maladaptation or algorithm aversion. For the same reason, attenuation of a trust reduction under rationale provision is not assumed to constitute improved calibration: explanations can support appropriate reliance when advice is sound but can also sustain reliance in incorrect advice (Cecil et al., 2024; Prinster et al., 2024; Schoeffer et al., 2024).

### 2.3 Self-efficacy theory and its contextual extension

Bandura’s self-efficacy framework defines efficacy beliefs as judgments about one’s capability to organize and execute actions required for task performance, with implications for effort, persistence, and coping (Bandura, 2006). In educational and AI-supported work, this framework has been extended to context-specific beliefs about using, evaluating, and integrating AI-related information (Collie et al., 2024; Chiu et al., 2025; Bergdahl and Sjöberg, 2025). Within human-AI decision-making, collaborative decision-making self-efficacy therefore concerns whether an individual believes that they can evaluate discrepant information and reach a workable decision with AI support. The present studies test whether disagreement is associated with lower post-manipulation self-efficacy and whether that association is linked to self-reported adaptive responses. Because self-efficacy is measured rather than manipulated, the indirect pathway is interpreted as consistent with mediation rather than as definitive causal evidence. Moreover, a reduction in self-efficacy under uncertainty is not assumed to be intrinsically harmful; it may reflect warranted uncertainty as well as disruptive self-doubt. This interpretive symmetry is important when evaluating the resilience moderation tested in Study 3.

### 2.4 The moderating role of psychological resilience

Psychological resilience is commonly conceptualized as the capacity to maintain or regain adaptive functioning under adversity, stress, or uncertainty, although its operationalization varies across stable individual-difference, coping-capacity, and dynamic-process approaches. The Connor-Davidson framework treats resilience as a measurable capacity for coping with stress, and the CD-RISC-10 provides a psychometrically refined short form for assessing individual differences in that capacity (Connor and Davidson, 2003; Campbell-Sills and Stein, 2007). In the present research, resilience is operationalized narrowly as a premeasured individual-difference resource rather than as an immutable personality trait or a directly observed recovery process. In Study 3, CD-RISC-10 was administered before the experimental session and is used to test moderation of the association between condition and post-manipulation self-efficacy. Because self-efficacy is not measured both before and after the conflict manipulation, the design tests a between-condition buffering pattern rather than within-person decline, recovery speed, or a dynamic resilience trajectory. Attenuation of the self-efficacy difference is therefore interpreted descriptively; without objective decision accuracy, the study cannot determine whether greater maintained confidence represents adaptive coping or unwarranted confidence.

### 2.5 Research gaps and theoretical model

Taken together, the model specifies the experimental condition (0 = consistent, 1 = conflicting) as X; machine trust (M1) and collaborative decision-making self-efficacy (M2) as simultaneous, potentially correlated mediators; and the self-report collaborative adaptive-response scale as the primary Y. This model addresses a gap at the intersection of JAS-based advice taking, trust calibration, AI-related efficacy beliefs, and physical-education decision support, a domain that remains comparatively underrepresented in the broader human-AI evidence base (Bonaccio and Dalal, 2006; Vaccaro et al., 2024; Wang and Wang, 2024; Zhou et al., 2024). Directional Weight of Advice (WOA) is treated separately as an advice-taking indicator rather than as decision quality, and its analysis is contingent on a valid nonzero advice-minus-initial-judgment denominator. Psychological resilience moderates the X → M2 pathway in Study 3, whereas rationale provision moderates the X → M1 pathway in Study 4. AI adoption intention is an exploratory secondary outcome and is not used to support H1-H8a. The parallel specification tests simultaneous indirect paths but does not establish causal independence between the mediators or exclude plausible serial relations between them. Critically, because recommendation accuracy is not manipulated or objectively established, the model identifies psychological response patterns but does not identify whether smaller reductions in trust or self-efficacy constitute better calibration or better decisions.

Based on the theoretical review and integrative model presented above, the following hypotheses are proposed:

*H1*: Machine trust will be significantly lower under the conflicting condition than under the consistent condition.

*H2*: Machine trust will carry a significant indirect effect of the experimental condition (0 = consistent, 1 = conflicting) on collaborative adaptive responses.

*H3*: Collaborative decision-making self-efficacy will be significantly lower under the conflicting condition than under the consistent condition.

*H4*: Collaborative decision-making self-efficacy will carry a significant indirect effect of the experimental condition on collaborative adaptive responses.

*H5*: Machine trust and collaborative decision-making self-efficacy will constitute two simultaneous indirect pathways between the experimental condition and collaborative adaptive responses; this parallel specification does not imply causal independence between the mediators.

*H6*: Psychological resilience will moderate the effect of the experimental condition on collaborative decision-making self-efficacy, such that the negative association between conflict and self-efficacy will be weaker at higher levels of resilience.

*H7*: Psychological resilience will moderate the conditional indirect effect of the experimental condition on collaborative adaptive responses through self-efficacy, such that the negative indirect effect will become less negative as resilience increases.

*H8a*: Rationale provision will moderate the effect of the experimental condition on machine trust, such that the negative effect of conflict will be weaker when a rationale is provided than in the black-box condition.

Study 4 scope: H8a is the only hypothesis evaluated in Study 4. A rationale-moderated indirect effect of experimental condition on collaborative adaptive responses through machine trust is not hypothesized or inferred because the required outcome-level conditional-process test is not reported; this remains a question for future research.

Interpretive boundary for H6-H8a. These hypotheses concern statistical attenuation of between-condition associations. They do not presuppose that higher retained trust or self-efficacy is beneficial. In the absence of objectively verified recommendation accuracy, the present design cannot distinguish appropriate calibration from overreliance, or warranted uncertainty from unwarranted confidence.

Figure 1 the cross-study conceptual model specifies conflict-related reductions in machine trust (H1) and collaborative decision-making self-efficacy (H3), with both mediators transmitting negative indirect effects to collaborative adaptive behavior (H2 and H4) within the parallel specification (H5). Psychological resilience moderates the condition-to-self-efficacy pathway in Study 3 (H6-H7), whereas rationale provision moderates the condition-to-trust pathway in Study 4 (H8a). No rationale-moderated indirect-effect hypothesis through machine trust is included because Study 4 does not report that conditional-process test. Because the moderators were examined in different study datasets, the diagram represents conceptual synthesis rather than a jointly estimated dual-moderation model; AI adoption intention remains an unconnected exploratory outcome.

**Figure 1:**
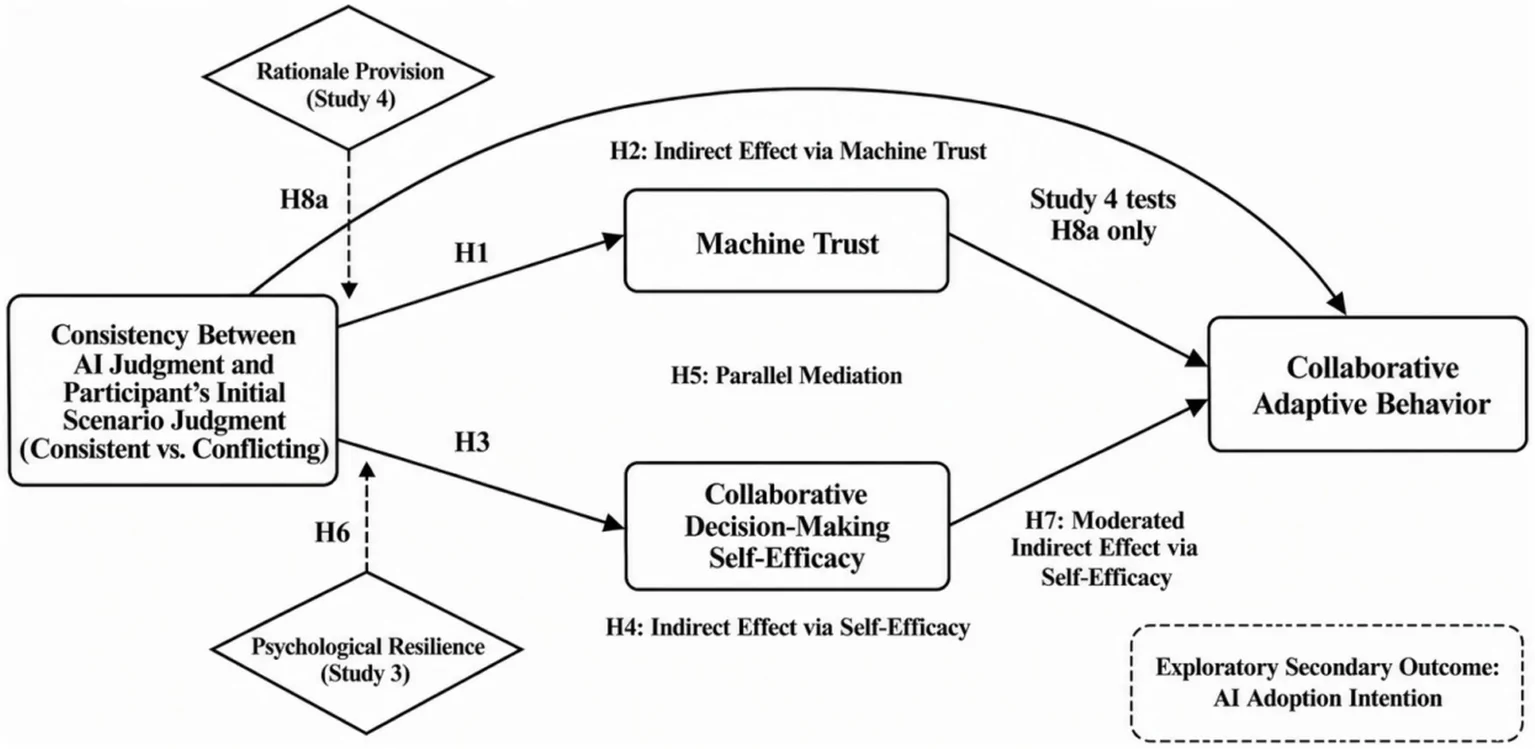
Cross-study conceptual model of simultaneous and study-specific moderated indirect pathways.

## 3 Methods

### 3.1 Study design

This research investigates whether machine trust and human-AI collaborative decision-making self-efficacy form simultaneous indirect pathways between the experimental consistency condition and subsequent collaborative adaptive responses, and whether psychological resilience and rationale provision serve as study-specific boundary conditions for the self-efficacy and trust pathways. The four experiments form an accumulating evidence sequence; results from different study datasets are not treated as a single estimated dual-moderation model.

Study 1 used a single-factor between-subjects design (consistent vs. conflicting) to test the basic trust-mediated pathway. Study 2 changed the scenario and added collaborative decision-making self-efficacy as a simultaneous mediator, providing a cross-situational extension rather than an independence-based replication claim. Study 3 used a two-wave, temporally separated design in which psychological resilience was measured at least 48 h before the formal experiment; because the same outcome was not measured at both waves, this was not a pretest-posttest design. Study 4 used a 2 (condition: consistent/conflicting) × 2 (rationale provision: black-box/rationale provided) between-subjects factorial design to test moderation of the trust response. The Study 3 and Study 4 moderator findings are synthesized conceptually across study-specific datasets.

### 3.2 Participants and sampling

Participants were required to have at least one year of teaching or coaching experience in physical education, including K-12 physical education teachers, university-level physical education instructors, sport-specific coaches, and senior undergraduate or graduate physical education majors with at least one cumulative year of internship or part-time coaching experience. Recruitment was conducted through teacher continuing-education platforms, partnerships with physical education departments, and professional associations, combining both online and offline channels. Eligibility screening items were used to verify the minimum experience requirement.

The analytic population comprised the mixed physical-education sample defined above, including in-service educators, coaches, and senior students with qualifying experience. Accordingly, population-level interpretations are limited to this mixed eligible sample and are not generalized exclusively to experienced in-service teachers.

Recruitment targets were prespecified as 180 participants for Study 1, 200 for Study 2, 280 for Study 3, and 260 for Study 4 (Table 1), with allowance for exclusions. These targets were operational recruitment goals rather than values derived from formal prospective power calculations, and the manuscript therefore does not retrospectively describe the studies as *a priori* powered. As sensitivity benchmarks at two-sided *α* = 0.05 and 1 − *β* = 0.80, the Study 1 allocation of 84 participants per condition corresponds to a detectable independent-groups standardized mean difference of approximately *d* = 0.435; Study 2 (*N* = 187) corresponds to an approximate two-group detectable contrast of *d* ≈ 0.41; the Study 3 one-degree-of-freedom interaction corresponds to approximately *f*^2^ = 0.030 (partial *R*^2^ ≈ 0.029); and the Study 4 factorial interaction corresponds to approximately *f*^2^ = 0.033 (partial η^2^ ≈ 0.032). To characterize the precision of the reported indirect effects without presenting a retrospective analysis as prospective power, a Monte Carlo sensitivity analysis was also conducted using 100,000 draws, two-sided α = 0.05, and random seed 1,972,538, parameterized by the reported indirect-effect estimates and bootstrap standard errors. The resulting approximate detection probabilities were.9997 for the Study 1 trust indirect effect, 0.9937 for the Study 2 trust indirect effect, 0.9162 for the Study 2 self-efficacy indirect effect, and.7997 for the Study 3 index of moderated mediation. These values are sensitivity diagnostics based on the reported effect estimates and their sampling uncertainty; they are not interpreted as evidence that the studies were prospectively powered for mediation or moderated mediation.

**Table 1:** Planned sample size, design type, and participant composition across studies.

Study	Planned *N*	Design	Participant composition	Primary test target
Study 1	180	Single-factor, two-group (between-subjects)	K-12/university teachers, coaches, senior PE majors	X → M1 → Y basic mediation
Study 2	200	Single-factor, two-group (between-subjects)	Same as above, new scenario	Parallel mediation (M1, M2)
Study 3	280	Single-factor, two-group + two-wave temporal separation	Same as above; resilience measured at Time 1	W moderates X → M2
Study 4	260	2 × 2 between-subjects factorial	Same as above; rationale-provision manipulation added	Rationale provision moderates X → M1

#### 3.2.1 Participant flow and exclusions

The reported participant-flow information is summarized in Table 2. Study 1 retained 168 participants (84 consistent, 84 conflicting). In Study 2, 200 cases entered the study flow; 13 were excluded during data-quality screening, leaving 187 participants for analysis (93 consistent, 94 conflicting). In Study 3, 280 participants completed Time 1; 19 were lost before Time 2 completion, yielding a final analytic sample of 261 participants (131 consistent, 130 conflicting). Study 4 retained 243 participants across the four factorial cells (61 consistent/black-box, 61 conflicting/black-box, 61 consistent/rationale, and 60 conflicting/rationale). The retained participant-level analysis files do not contain the complete pre-analysis screening history for Studies 1 and 4, the reason-by-condition distribution of the Study 2 exclusions, or a reason-by-condition account of Study 3 attrition. Accordingly, those quantities are reported only where they can be verified directly, and all focal analyses refer to the final complete-case/per-protocol samples.

**Table 2:** Available participant flow and final analytic allocation across studies.

Study	Flow start	Loss before final analysis	Final *N*	Final analytic allocation
Study 1	Not reconstructible from retained analytic files	Not reconstructible from retained analytic files	168	84 consistent; 84 conflicting
Study 2	200 entered study flow	13 data-quality exclusions	187	93 consistent; 94 conflicting
Study 3	280 completed Time 1	19 Time 1 → Time 2 attrition	261	131 consistent; 130 conflicting
Study 4	Not reconstructible from retained analytic files	Not reconstructible from retained analytic files	243	61 C/BB; 61 X/BB; 61\u00B0C/R; 60 X/R

C, consistent; X, conflicting; BB, black-box; R, rationale provided. “Not reconstructible from retained analytic files” indicates that the preserved de-identified final analysis files do not contain the corresponding pre-analysis screening/audit trail; these quantities are not inferred from planned recruitment targets.

#### 3.2.2 Data collection and record provenance

The corresponding author was responsible for the investigation, data curation, formal analysis, and project administration. Recruitment and study administration used the teacher continuing-education platforms, partner physical-education departments, and professional associations described above, with both online and offline channels. The preserved research files available for analysis are de-identified final analytic participant-level datasets rather than complete platform-level administrative audit trails. They retain the variables used in the reported analyses and the final condition labels, but they do not retain the full invitation/screening logs for Studies 1 and 4, the sequence-generation or allocation log needed to identify the original randomization routine, the original numerical attention/response-latency thresholds, or condition-linked exclusion-reason records. These historical operational details therefore cannot be reconstructed from the retained analytic files without introducing assumptions that are not documented in the preserved record. Accordingly, consistent with recommendations for transparent reporting of sample-size determination, data exclusions, experimental manipulations, and measures (Simmons et al., 2012), the manuscript reports only information that can be verified from the preserved records and explicitly identifies procedural information that is unavailable. Tables 3, 4 summarize the corresponding study materials and measurement information.

**Table 3:** Overview of scenario materials across studies.

Study	Scenario theme	Manipulated factor(s)	Calibration basis
Study 1	Sports injury risk alert	Experimental condition (consistent/conflicting)	Practitioner credibility review + pilot discrepancy calibration
Study 2	Training load/tactical adjustment	Experimental condition (consistent/conflicting)	Same calibration approach
Study 3	Movement technique error diagnosis	Experimental condition (consistent/conflicting)	Same calibration approach
Study 4	Integrated training decision	Experimental condition × Rationale provision (2 × 2)	Same calibration approach + rationale-provision check

**Table 4:** Study-specific measurement administration matrix.

Study	Time point	Instruments/variables explicitly reported	Administration order/reporting status
Study 1	Single session	Initial judgment; AI recommendation; final judgment/WOA; machine trust; collaborative adaptive behavior	Initial judgment → AI recommendation → final judgment; trust and adaptive-response measures were administered after the manipulation.
Study 2	Single session	Initial judgment; AI recommendation; final judgment/WOA; machine trust; collaborative decision-making self-efficacy; collaborative adaptive behavior	JAS sequence as above; trust and self-efficacy presentation order was randomized, and adaptive response was assessed post-manipulation.
Study 3	Time 1	CD-RISC-10 psychological resilience; demographics	Administered at least 48 h before the Time 2 experiment.
Study 3	Time 2	JAS sequence; machine trust; collaborative decision-making self-efficacy; collaborative adaptive behavior	Trust and self-efficacy presentation order was randomized; adaptive response was assessed post-manipulation.
Study 4	Single session	JAS sequence; rationale-provision manipulation; machine trust	2 × 2 condition × rationale procedure; machine trust was the focal Study 4 outcome used for H8a.
Exploratory project measure	Study-specific timing not documented in retained materials	AI adoption intention (4-item exploratory scale)	Disclosed for transparency; not assigned to a study without supporting records and excluded from focal psychometric and hypothesis-testing analyses.

This matrix summarizes the study-specific variables used in the focal analyses and the timing information supported by the retained study materials. AI adoption intention is disclosed as an exploratory secondary measure. Its study-specific administration information is not documented in the retained study materials; therefore it is excluded from study-specific psychometric, mediation, moderation, and causal claims rather than assigned to a particular study without supporting records.

#### 3.2.3 Allocation

Studies 1–3 used two-group between-subjects allocation structures, and Study 4 used a four-cell 2 × 2 between-subjects allocation structure. Final analytic allocations were 84/84 in Study 1, 93/94 in Study 2, 131/130 in Study 3, and 61/61/61/60 across the four Study 4 cells. The retained analytic files record final condition labels but do not contain the sequence-generation or assignment log needed to determine whether the original routine was simple, blocked, stratified, or used another algorithmic procedure. The manuscript therefore reports the observed allocations without assigning an unverified randomization mechanism. Study-level demographic summaries in Table 5 are descriptive and are not presented as substitutes for condition-specific baseline-equivalence tests.

**Table 5:** Demographic characteristics of final samples across the four studies.

Variable	Study 1 (*n* = 168)	Study 2 (*n* = 187)	Study 3 (*n* = 261)	Study 4 (*n* = 243)
Male (%)	54.2	51.3	49.8	52.7
Age, M (SD)	33.6 (7.2)	32.9 (6.8)	34.1 (7.5)	33.4 (7.1)
Years of teaching/coaching, M (SD)	7.8 (5.1)	7.3 (4.9)	8.1 (5.4)	7.6 (5.0)
K-12 teacher (%)	42.3	39.6	41	40.3
University instructor (%)	21.4	23	20.7	22.2
Sport-specific coach (%)	19.6	18.7	20.3	19.8
Senior PE major (%)	16.7	18.7	18	17.7
AI familiarity (1–7), M (SD)	3.85 (1.24)	3.92 (1.31)	3.78 (1.29)	3.96 (1.22)

Multiplicity and inferential scope. The hypotheses are evaluated within study-specific models rather than combined into a single pooled significance test, and the manuscript does not use cross-study *p*-value aggregation. No multiplicity-adjustment procedure was applied; *p* values are therefore interpreted as nominal and are considered together with effect sizes and confidence intervals. The exploratory AI-adoption outcome is not used to support H1–H8a, and no claim of family-wise or false-discovery-rate control is made.

Preregistration and analytic disclosure. None of the four studies is presented as preregistered, and no preregistration identifier or registered analysis plan is claimed. The theoretically specified H1–H8a tests are distinguished from the exploratory AI-adoption outcome, and the absence of preregistration is taken into account when characterizing the confirmatory strength of the evidence.

### 3.3 Scenario development, credibility review, and calibration

All studies employed scenario-based materials adapted from the JAS paradigm (Bonaccio and Dalal, 2006; Harvey and Fischer, 1997). Participants first rendered an initial judgment and then received an experimentally presented AI recommendation assigned to the consistent or conflicting condition relative to that judgment. The manipulation therefore operationalizes advice-judgment agreement rather than a direct comparison of data-driven and experiential judgment. Pretesting targeted an approximately two-point discrepancy for the conflicting condition as a calibration goal, not as a claim that every participant received a fixed ±2-point arithmetic offset. Because the study protocol records do not provide one deterministic participant-level mapping or endpoint rule, the manipulation is reported at the categorical condition level. The manuscript does not infer a participant-specific numerical yoking rule that is not documented in the study records.

Scenario materials underwent practitioner credibility review and informal pretesting before formal data collection. The practitioner review is treated as a qualitative face-validity check rather than as a formal psychometric validation exercise. The study records do not provide a complete reviewer roster, standardized rating instrument, inter-rater statistic, or participant-level pretesting flow; therefore no numerical panel or pretesting statistics are reported. Pretesting observations were used only to refine scenario plausibility and discrepancy strength and were not included in the analytic samples. A formal cross-scenario matching test on stakes, ambiguity, and time pressure was not conducted, so these dimensions remain limitations of cross-study comparison. Full scenario texts in the original language of administration and translated versions are included in the Supplementary materials.

### 3.4 Measures

Machine trust was assessed with a six-item context-adapted human-automation trust scale (Jian et al., 2000), collaborative decision-making self-efficacy with a seven-item scale grounded in Bandura’s efficacy framework (Bandura, 2006; Collie et al., 2024), psychological resilience with the 10-item Chinese CD-RISC (Connor and Davidson, 2003; Campbell-Sills and Stein, 2007), and collaborative adaptive behavior with a five-item self-report scale. A four-item AI adoption-intention measure formed part of the exploratory project materials; because its study-specific administration information is not documented in the retained study materials, it is disclosed for transparency but is not assigned to a particular study without supporting records or analyzed in the focal hypothesis tests or study-specific psychometric models. The Supplementary materials provide item wording, response anchors, scoring rules, and translation/adaptation information for the focal measures. Directional WOA is defined as (final judgment—initial judgment)/(AI recommendation—initial judgment) and is treated as a descriptive advice-taking indicator rather than as objective decision quality. Teaching experience, educational stage, AI familiarity, digital literacy, and general trust propensity were recorded as candidate covariates; the focal results are presented without covariate adjustment.

### 3.5 Research ethics and debriefing

All participants provided informed consent before participation. The AI feedback presented in the JAS procedure was an experimental manipulation rather than genuine system output. Participants received an immediate debriefing after each study that disclosed the purpose and nature of the manipulation.

### 3.6 Study 1 design and analytic

Study 1 used a single-factor between-subjects design with a final analytic sample of 168 participants (84 per condition) to test H1 and H2. Experimental condition was dummy coded 0 = consistent and 1 = conflicting. Unadjusted independent-samples t tests were used for the manipulation and trust comparisons, and PROCESS Model 4 with 5,000 bootstrap resamples was used for the indirect effect (Hayes, 2022). The reported primary results are unadjusted. Because the manipulation check preceded the outcomes, possible demand awareness is treated as a design limitation.

### 3.7 Study 2 design and analytic

Study 2 used the single-factor between-subjects design and included a final analytic sample of 187 participants (93 consistent, 94 conflicting). Thirteen cases were excluded during data-quality screening before the final analytic sample was defined. PROCESS Model 4 estimated trust and self-efficacy as simultaneous, correlated mediators and reported the total effect, direct effect, each specific indirect effect, and their contrast. The condition-to-self-efficacy path (*B* = −0.687, SE = 0.132, *p* < 0.001) provides the direct test of H3. The parallel specification does not establish statistical or causal independence between the mediators.

### 3.8 Study 3 design and analytic

Study 3 used a two-wave, temporally separated design. A total of 280 participants completed Time 1, at which psychological resilience and demographics were measured; 19 participants were lost before Time 2 completion, leaving a final analytic sample of 261 participants (131 consistent, 130 conflicting). The JAS experiment was completed at Time 2. This design tests whether resilience moderates the X → M2 path; it does not estimate within-person decline or recovery. The first-stage conditional-process model used 5,000 bootstrap resamples. The self-efficacy equation included condition, resilience, and their interaction, whereas the adaptive-behavior equation included condition, self-efficacy, and machine trust. Results are reported as observed-variable regression coefficients rather than as a jointly estimated dual-moderation system.

### 3.9 Study 4 design and analytic

Study 4 used a 2 (condition: consistent/conflicting) × 2 (rationale provision: black-box/rationale provided) between-subjects factorial design with a final analytic sample of 243 participants. The factorial ANOVA tested the condition × rationale-provision interaction on machine trust, followed by simple-effects comparisons. Because the rationale-provided condition added explanatory information that was absent from the black-box condition, the inference is limited to the effect of providing a rationale rather than explainability in general (Humer et al., 2024). Resilience was not included in the Study 4 model, and Study 4 is not combined with Study 3 as a single dual-moderation analysis. Study 4 therefore evaluates H8a only; no moderated-mediation hypothesis through machine trust is advanced in this study.

Figure 2A the design matrix separates experimental consistency from rationale provision. All four conditions received an AI judgment outcome, whereas only the two rationale-provided conditions received an accompanying explanation. This symmetric presentation preserves the orthogonality of the 2 × 2 manipulation and prevents information availability from being confounded with consistency.

**Figure 2:**
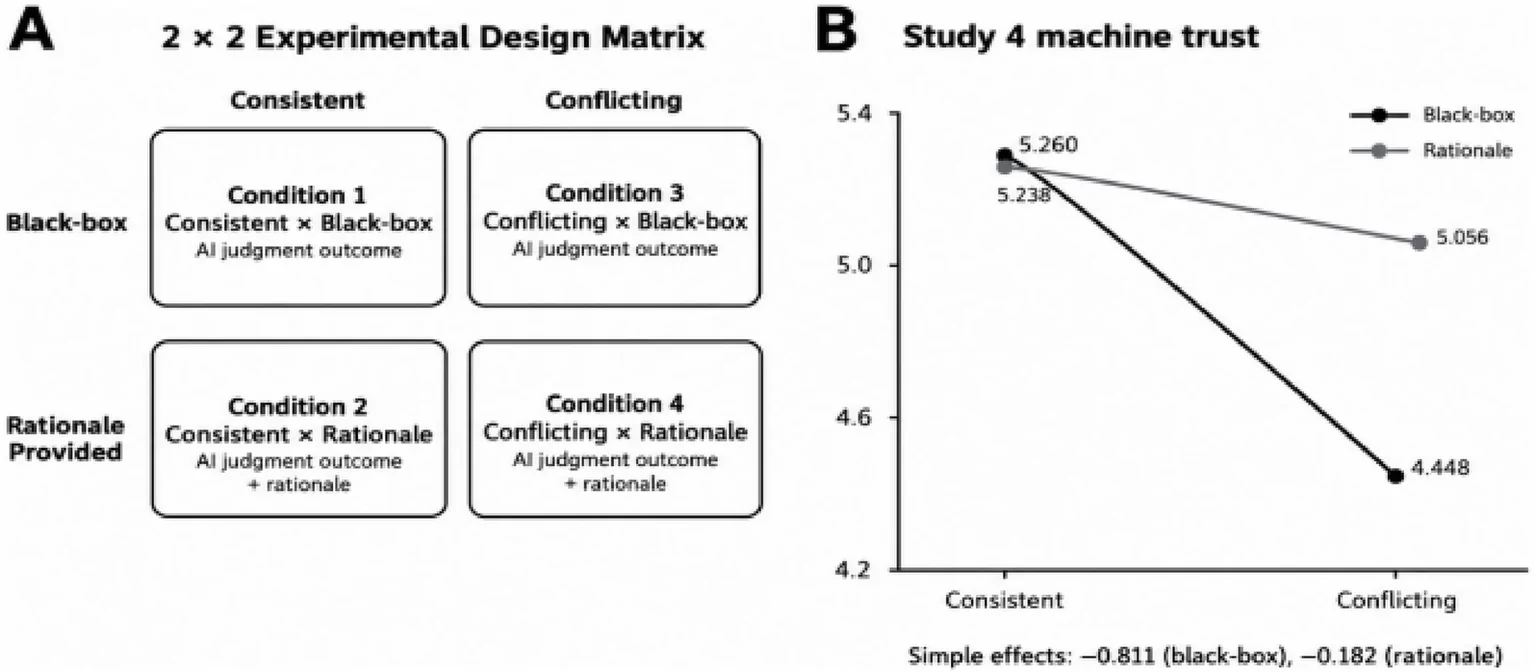
Study 4 design and trust response: **(A)** 2 × 2 experimental design matrix; **(B)** Between-condition trust means. Simple effects: −0.811 (black-box), −0.182 (rationale).

Figure 2B panel B represents between-condition machine-trust means rather than a within-person change score because Study 4 did not include a pre-manipulation trust measure. The four cell means (SDs) are 5.260 (0.69) for consistent/black-box, 4.448 (0.72) for conflicting/black-box, 5.238 (0.76) for consistent/rationale, and 5.056 (0.72) for conflicting/rationale. The corresponding conflict effects are −0.811 in the black-box condition and −0.182 in the rationale-provided condition, consistent with the condition × rationale interaction, *F*(1, 239) = 11.348, *p* < 0.001.

### 3.10 Common method bias and data quality control

The presentation order of the trust and self-efficacy measures was randomized. Harman’s single-factor test was treated only as a descriptive diagnostic and is not used to claim that common-method variance was absent. Data-quality screening included attention and response-latency checks before analysis (Cuskley and Sulik, 2024). The preserved final analytic files contain the retained cases but do not preserve the original screening configuration or an auditable condition-linked exclusion log. Consequently, the aggregate Study 2 exclusion count is reported, but the historical numerical cutoffs and reason-by-condition screening detail are not reconstructed from the final dataset. The focal indirect-effect analyses use the stated PROCESS specifications and 5,000-resample bootstrap procedure, and primary results are presented without covariate adjustment. This distinction between final analytic data and missing administrative screening metadata is treated as a procedural reproducibility limitation rather than replaced by assumed thresholds.

## 4 Results

### 4.1 Data cleaning and sample description

After application of the stated quality-control criteria, the reported complete-case/per-protocol samples were 168 for Study 1, 187 for Study 2, 261 for Study 3, and 243 for Study 4. Study 2 excluded 13 cases from the 200-case study flow, producing final condition counts of 93 consistent and 94 conflicting. Study 3 included 261 of the 280 Time 1 participants after 19 Time 1-to-Time 2 attrition cases, producing final condition counts of 131 consistent and 130 conflicting. The results therefore apply to the retained analytic samples. Exclusions and attrition are not interpreted as missing completely at random, and no intention-to-treat inference is made.

Demographic characteristics are summarized in Table 5. The four samples were descriptively similar in aggregate composition, but similarity of marginal percentages does not establish cross-study equivalence or measurement invariance. Because the samples combine in-service teachers, instructors, coaches, and senior students, the findings are interpreted for this mixed eligible population rather than generalized specifically to experienced teachers.

### 4.2 Reported reliability and discriminant validity

Measurement quality is reported separately for the sample in which each construct was administered. Table 6 presents study-specific Cronbach’s α, composite reliability (CR), and average variance extracted (AVE) values aligned with the study-specific measurement structure and analytic Ns.

**Table 6:** Study-specific reliability and convergent validity.

Study/construct	Reported analytic *N*	Cronbach’s α	CR	AVE
Study 1/machine trust	168	0.895	0.919	0.656
Study 1/adaptive behavior	168	0.842	0.889	0.615
Study 2/machine trust	187	0.885	0.912	0.635
Study 2/self-efficacy	187	0.878	0.906	0.579
Study 2/adaptive behavior	187	0.838	0.885	0.607
Study 3/machine trust	261	0.860	0.896	0.589
Study 3/self-efficacy	261	0.901	0.922	0.627
Study 3/psychological resilience	261	0.874	0.898	0.470
Study 3/adaptive behavior	261	0.877	0.911	0.671
Study 4/machine trust	243	0.767	0.838	0.463

Because no single study contained all five constructs, Table 7 reports study-specific construct correlations and √AVE comparisons for the construct sets jointly measured in Studies 1–3. Study 4 included machine trust as the focal measured construct for H8a, so a multiconstruct discriminant-validity comparison is not applicable there. No pooled measurement model is interpreted across studies.

**Table 7:** Study-specific discriminant validity.

Study	Construct set jointly reported	Reported analytic *N*	Correlation matrix	√AVE comparisons	Applicability
Study 1	T; A	168	r(T, A) = 0.532	√AVE T = 0.810; A = 0.784	Study-specific
Study 2	T; S; A	187	r(T, S) = 0.538 r(T, A) = 0.389 r(S, A) = 0.358	√AVE T = 0.797 S = 0.761; A = 0.779	Study-specific
Study 3	T; S; R; A	261	r(T, S) = 0.460 r(T, R) = 0.160 r(T, A) = 0.505 r(S, R) = 0.432 r(S, A) = 0.550 r(R, A) = 0.229	√AVE T = 0.767 S = 0.792; R = 0.686 A = 0.819	Study-specific
Study 4	T only	243	Only trust available	Not applicable	Not applicable

T, machine trust; S, self-efficacy; R, resilience; A, adaptive behavior.

### 4.3 Study 1 results

Using the reported group sizes, means, and standard deviations, the manipulation check showed that participants in the conflicting condition rated agreement between the AI judgment and their initial scenario judgment (*M* = 2.86, SD = 1.14) lower than participants in the consistent condition (*M* = 5.63, SD = 0.97), *t*(166) = 16.96, *p* < 0.001.

Table 8 presents descriptive statistics and between-group comparisons for the focal Study 1 variables. Machine trust was significantly lower in the conflicting condition than in the consistent condition, *t*(166) = 6.87, *p* < 0.001, Cohen’s *d* = 1.061, supporting H1, and collaborative adaptive behavior was likewise lower under conflict. WOA was higher in the consistent condition (M = 0.619, SD = 0.191) than in the conflicting condition (*M* = 0.478, SD = 0.205), *t*(166) = 4.61, *p* < 0.001, *d* = 0.711. This WOA contrast is interpreted as advice taking rather than as evidence of superior decision quality.

**Table 8:** Descriptive statistics and between-group comparisons in study 1.

Variable	Consistent (*n* = 84) M(SD)	Conflicting (*n* = 84) M(SD)	*t*	*p*	Cohen’s d
Machine trust	5.42 (0.98)	4.31 (1.12)	6.87	<0.001	1.061
Collaborative adaptive behavior	4.78 (0.87)	4.15 (0.93)	4.62	<0.001	0.698
Weight of advice (WOA)	0.619 (0.191)	0.478 (0.205)	4.61	<0.001	0.711

With experimental condition coded 0 = consistent and 1 = conflicting, conflict negatively predicted machine trust (a = −1.108, SE = 0.161, *p* < 0.001), and machine trust positively predicted collaborative adaptive responses (*b* = 0.386, SE = 0.058, *p* < 0.001). Their product yielded a negative indirect effect, B = −0.428 (Boot SE = 0.079, 95% bootstrap CI [−0.591, −0.286]), supporting H2. The direct effect remained negative (c′ = −0.201, SE = 0.064, *p* = 0.002), and the unadjusted total effect was c = −0.629 (SE = 0.136, *p* < 0.001), satisfying c = c′ + ab. The indirect effect represented approximately 68.0% of the total effect in absolute magnitude.

Figure 3 with condition coded 0 = consistent and 1 = conflicting, conflict predicted lower machine trust (*a* = −1.108), whereas trust positively predicted collaborative adaptive behavior (b = 0.386). Their product yielded a negative indirect effect [ab = −0.428, 95% bootstrap CI (−0.591, −0.286)], supporting H2. The direct effect remained negative (c′ = −0.201, *p* = 0.002), and the unadjusted total effect was c = −0.629; thus, c = c′ + ab and the pattern indicates partial mediation rather than complete mediation.

**Figure 3:**
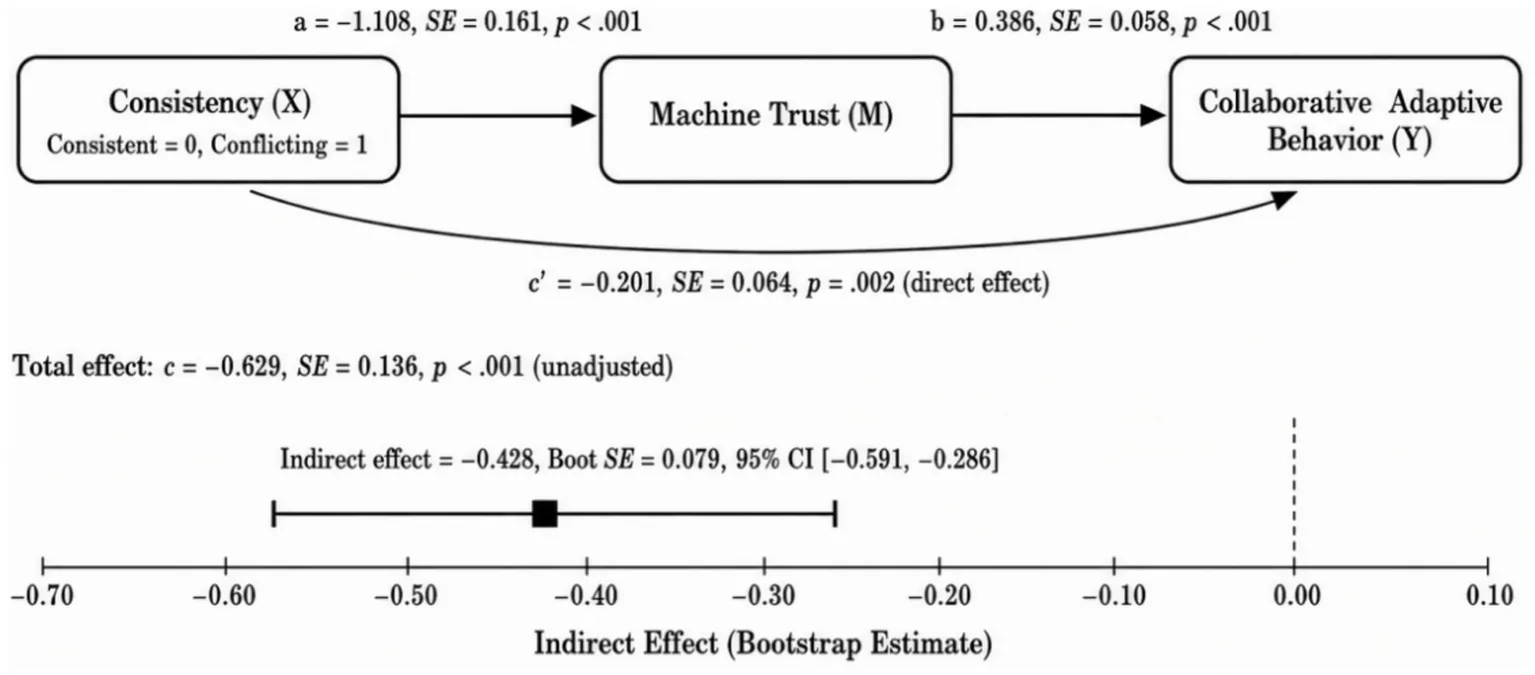
Study 1 single-mediator model through machine trust. Path diagram of the study 1 single-mediator model. Values are unstandardized coefficients; c’ denotes the direct effect and c denotes the total effect. With consistent = 0 and conflicting = 1, c = c’ + ab = −0.201 + (−0.428) = −0.629. The total-effect *SE* is the unadjusted value implied by Table 8. The bottom axis displays the bootstrap indirect effect with its 95% confidence level.

### 4.4 Study 2 results

The manipulation check remained effective following the scenario change, *t*(185) = 16.47, *p* < 0.001. With the common coding 0 = consistent and 1 = conflicting, the total effect was B = −0.512 [SE = 0.071, 95% CI (−0.652, −0.372)] and the direct effect was B = −0.184 [SE = 0.068, 95% CI (−0.317, −0.051)], indicating partial mediation Table 9.

**Table 9:** Decomposition of parallel mediation effects in study 2.

Effect	Point estimate	SE	Boot 95% CI LL	Boot 95% CI UL
Total effect	−0.512	0.071	−0.652	−0.372
Direct effect	−0.184	0.068	−0.317	−0.051
Indirect effect via trust	−0.201	0.045	−0.296	−0.118
Indirect effect via self-efficacy	−0.127	0.038	−0.207	−0.058
Contrast (trust vs. self-efficacy)	−0.074	0.052	−0.176	0.021

Conflict significantly reduced collaborative decision-making self-efficacy (B = −0.687, SE = 0.132, *p* < 0.001), supporting H3. The bootstrap confidence intervals for the specific indirect effects through machine trust [B = −0.201, 95% CI (−0.296, −0.118)] and self-efficacy [B = −0.127, 95% CI (−0.207, −0.058)] excluded zero, supporting H4 and the simultaneous-indirect-pathway claim in H5. The contrast between the two indirect effects was not significant [B = −0.074, 95% CI (−0.176, 0.021)]; this result indicates insufficient evidence of a difference and does not establish equivalence or independence between the mediators.

Figure 4 conflict reduced both machine trust (a1 = −1.042) and collaborative decision-making self-efficacy (a2 = −0.687), and both mediators were positively associated with collaborative adaptive behavior. The specific indirect effects through trust [B = −0.201, 95% CI (−0.296, −0.118)] and self-efficacy [B = −0.127, 95% CI (−0.207, −0.058)] were significant, producing a total indirect effect of −0.328. The remaining direct effect was −0.184, consistent with partial parallel mediation. The contrast interval [−0.176, 0.021] crossed zero, providing no evidence that the two indirect effects differed in magnitude; the negative condition-to-self-efficacy path supports H3.

**Figure 4:**
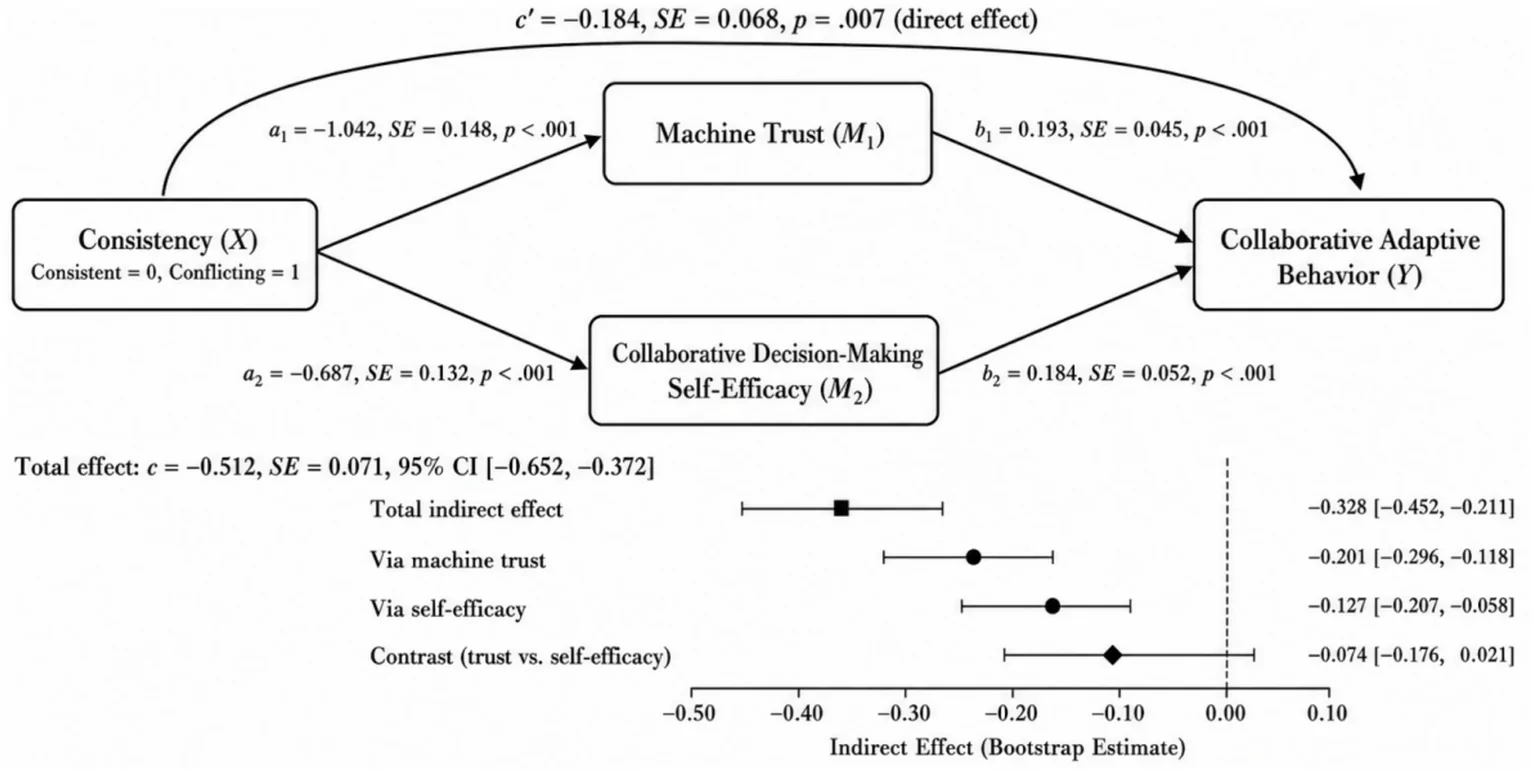
Study 2 parallel-mediation model through machine trust and collaborative decision-making self-efficacy. Path diagram of the Study 2 parallel-mediation model. Values are unstandardized coeffcients. Experimental condition was coded 0 = consistent and 1 = conflicting. The negative *X*→ self-efficacy path supports H3. The inset displays bootstrap indirect effects and their 95% confidence intervals; the contrast interval includes zero.

### 4.5 Study 3 results

Across the two-wave, temporally separated design, the mean interval was 52.6 h (SD = 6.8). With condition coded 0 = consistent and 1 = conflicting, conflict was associated with lower post-manipulation self-efficacy (B = −0.612, SE = 0.089, *p* < 0.001), resilience was positively associated with self-efficacy (B = 0.334, SE = 0.062, *p* < 0.001), and the positive condition × resilience interaction (B = 0.276, SE = 0.084, *p* = 0.001) indicated that the negative between-condition association became weaker as resilience increased. This supports H6 as a statistical attenuation pattern, not a claim about within-person decline or recovery and not evidence, by itself, that maintaining higher self-efficacy improves decision quality.

The index of moderated mediation was positive [index = 0.115, Boot SE = 0.041, 95% bootstrap CI (0.041, 0.203)]. Given the common coding and the positive self-efficacy-to-outcome coefficient, the negative indirect effect through self-efficacy became less negative as resilience increased, supporting H7. This moderated indirect effect describes how the measured response pattern varies with resilience; without objective ground truth it does not identify whether the attenuation is beneficial, neutral, or potentially associated with unwarranted confidence. The positive direct condition coefficient in the adaptive-response equation (B = 0.198, SE = 0.071, *p* < 0.01) is interpreted as a residual effect conditional on the mediators and not as evidence that conflict had a positive total effect Table 10.

**Table 10:** Regression results of the moderated mediation model in study 3.

Predictor	Self-efficacy model B(SE)	Adaptive behavior model B(SE)
Experimental condition (0 = consistent, 1 = conflicting)	−0.612 (0.089)***	0.198 (0.071)**
Resilience	0.334 (0.062)***	—
Experimental condition × resilience	0.276 (0.084)**	—
Self-efficacy	—	0.418 (0.067)***
Machine trust	—	0.352 (0.069)***
*R* ^2^	0.284	0.391

***p < 0.001, **p < 0.01. Experimental condition was coded 0 = consistent and 1 = conflicting. Index of moderated mediation = 0.115, Boot SE = 0.041, 95% bootstrap CI [0.041, 0.203]. The positive direct condition coefficient is interpreted as a residual effect conditional on the mediators rather than as the total effect of conflict.

### 4.6 Study 4 results

For Study 4 (*N* = 243), an effect-coded 2 × 2 ordinary-least-squares ANOVA yielded a condition main effect, *F*(1, 239) = 28.288, *p* < 0.001, partial η^2^ = 0.106; a rationale main effect, *F*(1, 239) = 9.826, *p* = 0.00194, partial η^2^ = 0.039; and a condition × rationale interaction, *F*(1, 239) = 11.348, *p* = 0.00088, partial η^2^ = 0.045. The common error mean square was 0.530 (SSerror = 126.677), (Table 11).

**Table 11:** Study 4 2 × 2 ANOVA results.

Source	df	*F*	*p*	Partial η^2^
Experimental condition	1	28.288	<0.001	0.106
Rationale provision	1	9.826	0.00194	0.039
Condition × rationale	1	11.348	0.00088	0.045
Error	239	—	—	—

The four-cell descriptive statistics were: consistent/black-box M = 5.260 (SD = 0.69, *n* = 61), conflicting/black-box M = 4.448 (SD = 0.72, *n* = 61), consistent/rationale M = 5.238 (SD = 0.76, *n* = 61), and conflicting/rationale M = 5.056 (SD = 0.72, *n* = 60). Using the common ANOVA error term, the conflict effect was −0.811 in the black-box condition (SE = 0.132, *t*(239) = −6.156, *p* < 0.001, 95% CI [−1.071, −0.552]) and −0.182 when a rationale was provided (SE = 0.132, *t*(239) = −1.376, *p* = 0.170, 95% CI [−0.443, 0.079]). The difference between these simple effects was compatible with the condition × rationale interaction, *F*(1, 239) = 11.348.

Figure 5A panel A separates the two study-specific boundary-condition models. In Study 3, resilience weakened the negative condition-to-self-efficacy association (interaction B = 0.276, *p* = 0.001), and the index of moderated mediation was positive [0.115, 95% bootstrap CI (0.041, 0.203)], supporting H6 and H7 as attenuation effects. In Study 4, rationale provision moderated the condition-to-trust association, *F*(1, 239) = 11.348, *p* < 0.001, partial η^2^ = 0.045, with a larger conflict-related trust reduction in the black-box condition than in the rationale-provided condition. Neither moderator is assigned a beneficial welfare interpretation because recommendation accuracy was not established, and the coefficients are not combined into a single dual-moderation model.

**Figure 5:**
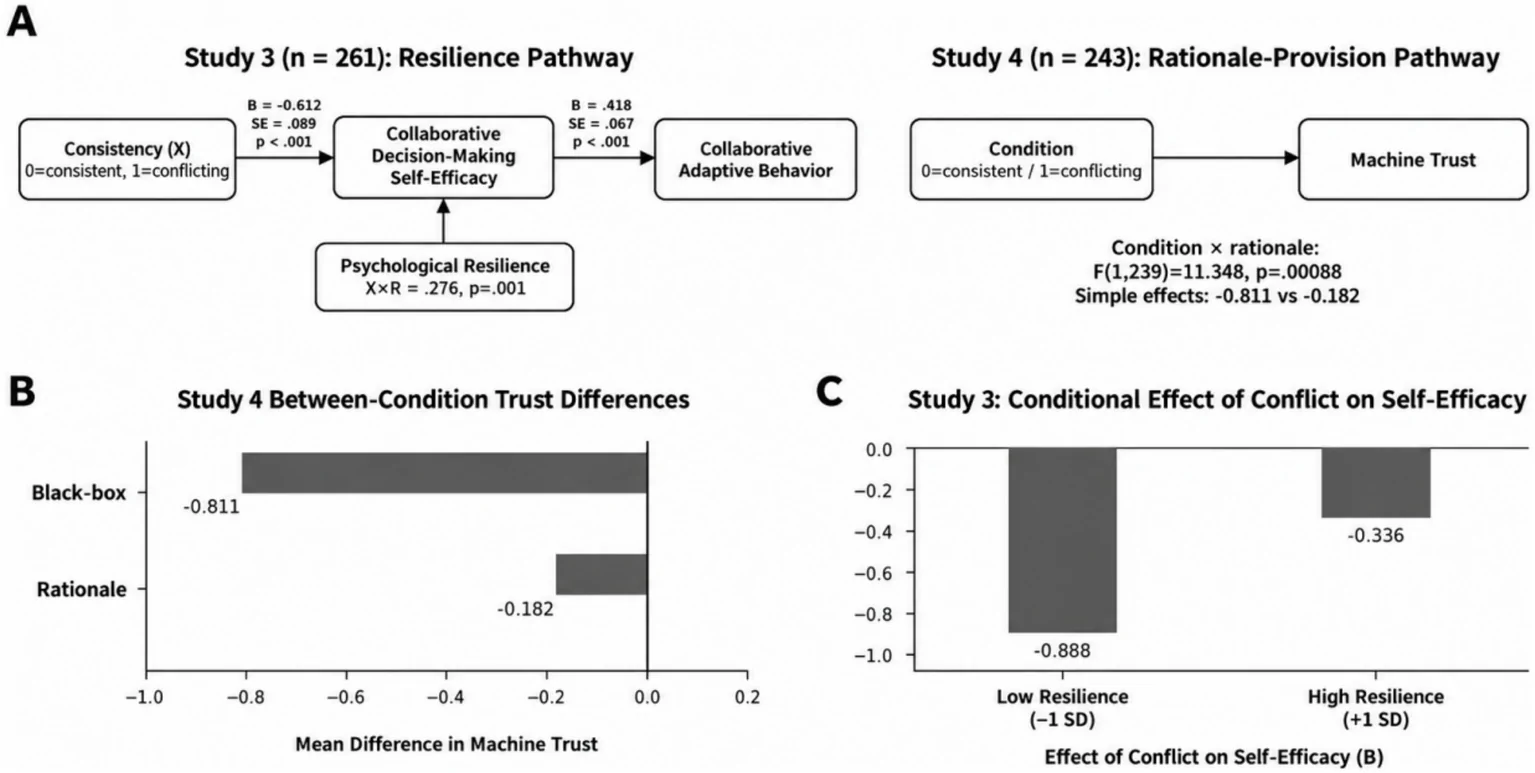
Cross-study conceptual synthesis of boundary conditions: **(A)** Study-specific selected paths; **(B)** Study 4 simple-effect comparison; **(C)** Study 3 conditional effects of conflict on self-efficacy.

Figure 5B the Study 4 panel represents between-condition trust differences, not within-person change. The conflict effects are −0.811 in the black-box condition and −0.182 in the rationale-provided condition, consistent with the condition × rationale interaction, *F* = 11.348.

Figure 5C the Study 3 conditional slopes quantify the resilience buffer. At low resilience (−1 SD), the estimated conflict effect on self-efficacy was −0.888; at high resilience (+1 SD), it was −0.336. The 0.552-point difference follows from the positive interaction coefficient (*B* = 0.276) across the two-standard-deviation contrast and indicates that resilience reduced, but did not reverse, the negative association between conflict and self-efficacy.

### 4.7 Cross-study descriptive integration

Across the four studies, the reported coefficients were directionally consistent after harmonizing the coding of experimental condition. Study 3 provided evidence that resilience moderated the self-efficacy pathway, whereas Study 4 showed that rationale provision moderated the trust pathway. These findings constitute accumulating evidence across study-specific datasets rather than a single empirically estimated dual-moderation model or an independence-based replication claim. This section is descriptive and is not presented as a robustness or sensitivity analysis. Figure 6 provides a descriptive, non-pooled comparison of the reported Study 1 and Study 2 indirect effects; it does not test effect equality, heterogeneity, robustness to covariate adjustment, or generalizability across studies.

**Figure 6:**
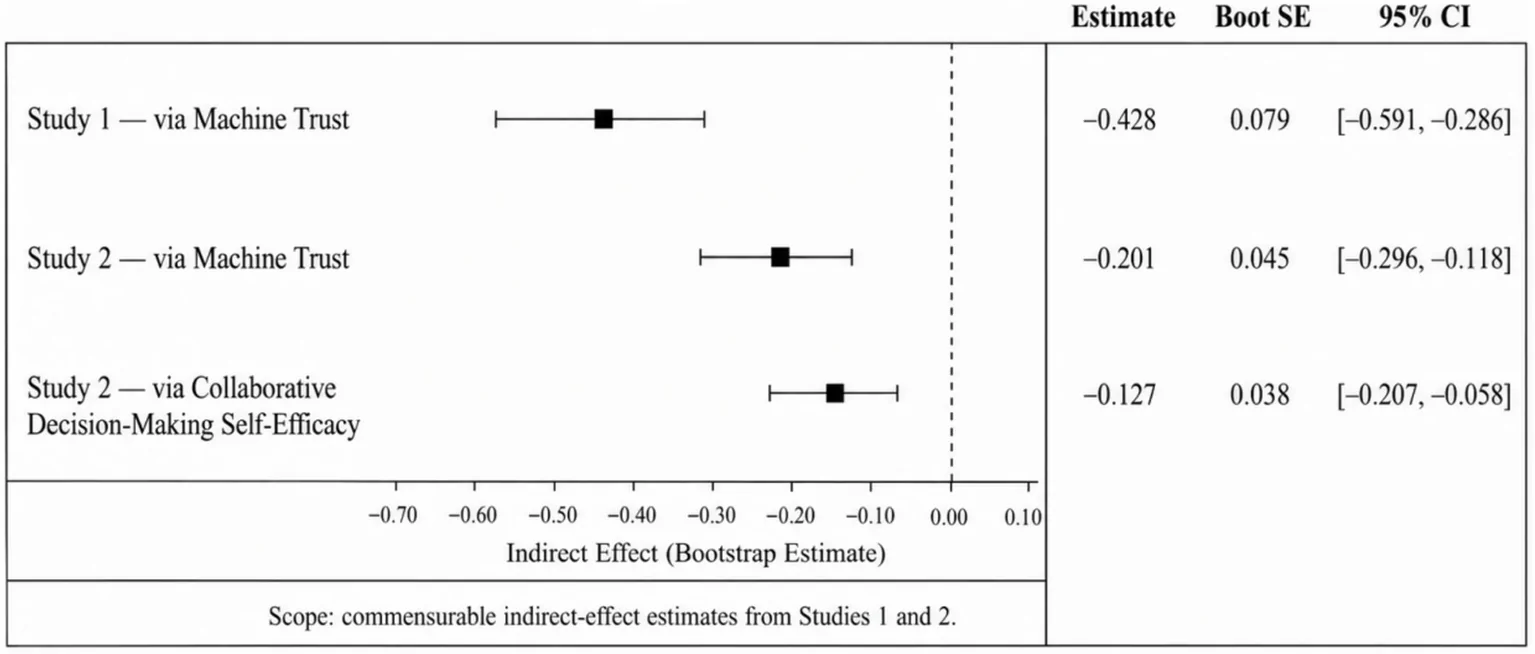
Descriptive, non-pooled forest plot of reported indirect effects in studies 1 and 2. Effects are shown descriptively under the common coding 0 = consistent and 1 = conflicting. No pooled estimate, meta-analytic weighting, or heterogeneity statistic was calculated. The plotted effects represent different study contexts and mediation estimands; the two Study 2 estimates are correlated because they come from the same sample. Equal-sized squares denote point estimates, horizontal lines denote 95% bootstrap confidence intervals, and the dashed vertical line denotes zero.

Figure 6 the descriptive forest plot shows three harmonized negative indirect effects whose bootstrap confidence intervals exclude zero: Study 1 through machine trust (*B* = −0.428), Study 2 through machine trust (*B* = −0.201), and Study 2 through self-efficacy (*B* = −0.127). The comparison is restricted to the commensurable indirect-effect estimates available for Studies 1 and 2. The estimates are not pooled because they represent different scenarios and mediation estimands, and the two Study 2 effects are correlated within the same sample; consequently, the figure does not imply cross-study homogeneity or meta-analytic generalizability.

## 5 Discussion

Across the four scenario-based experiments reported here, disagreement between AI-generated advice and participants’ own scenario judgments was associated with lower machine trust and lower collaborative decision-making self-efficacy, and the measured mediators carried negative indirect associations with collaborative adaptive responses (Cecil et al., 2024; Nagendran et al., 2024). Study 3 indicated that the condition-to-self-efficacy association was weaker at higher resilience, and Study 4 showed that rationale provision attenuated the condition-related difference in machine trust. These boundary conditions were examined in different studies rather than within one jointly estimated model. Importantly, the moderation patterns describe attenuation of psychological responses; they do not establish that preserving trust or self-efficacy is desirable. Because the scenarios did not establish objective recommendation accuracy, the same attenuation could reflect better calibration in some circumstances and overreliance or unwarranted confidence in others.

The WOA results are interpreted in the same non-normative manner. Study 1 showed higher advice taking in the consistent than in the conflicting condition. This difference is not treated as evidence of superior decision quality. Without objective ground truth, lower reliance under disagreement could reflect appropriate skepticism toward incorrect advice, resistance to correct advice, or a mixture of both. Accordingly, the study does not interpret reduced trust, reduced reliance, or their attenuation as evidence of decision safety.

That trust and self-efficacy appear to operate in parallel may point toward two distinct but related appraisal processes. Directed outward, trust reflects judgments about the system’s competence and reliability, and a decline under disagreement can be consistent with either rational recalibration or disagreement-based resistance (Visser et al., 2025). Directed inward, self-efficacy reflects a practitioner’s perceived capability to evaluate discrepant information and reach a workable decision; lower self-efficacy may signal disruptive self-doubt, but it may also represent warranted uncertainty when the evidence is ambiguous. The moderators should therefore be interpreted symmetrically. Rationale provision may help users maintain appropriately calibrated trust when advice is accurate, yet the same mechanism may sustain excessive trust when advice is inaccurate; likewise, resilience may preserve useful confidence or, under some conditions, unwarranted confidence. The present design cannot distinguish these possibilities because recommendation accuracy was not independently established (Cecil et al., 2024; Schoeffer et al., 2024).

Rather than forcing resilience and rationale provision into a single dual-moderation model, each was treated as a boundary condition specific to its own study. This preserves the study-specific estimands and avoids implying that the two moderators were jointly tested. At the same time, the use of different scenarios and study-specific datasets means that cross-study differences may reflect scenario, sample-composition, or manipulation-strength differences as well as moderation. Because participant overlap across studies cannot be established from the study records, the cross-study synthesis is descriptive rather than an independence-based replication claim. The resulting effect sizes should therefore be read as accumulating, context-bound evidence rather than components of one calibrated system. The same caution applies to trust: lower trust is treated as ambiguous between appropriate calibration and disagreement-based resistance, and attenuation of that reduction is equally ambiguous in normative terms (Visser et al., 2025).

Several constraints should be kept in view when weighing these conclusions. The studies relied on hypothetical scenarios rather than authentic high-stakes decisions, and predominantly self-report measures may not fully capture how conflict unfolds in live teaching or coaching practice (Shevtsova et al., 2024). With the exception of Study 3, data were gathered at a single time point, limiting inference about recovery or longer-term adaptation. The mixed sample of in-service teachers, coaches, instructors, and senior students broadens the eligible population but limits subgroup-specific generalization (van Arum et al., 2025). In addition, different scenarios were used across studies, so scenario is confounded with study and cross-situational consistency cannot be separated cleanly from sample and material differences. Most importantly, no objectively verifiable recommendation accuracy was established. Consequently, neither trust nor self-efficacy can be evaluated against an external criterion of appropriate reliance or decision correctness, and the welfare direction of the observed moderation effects is not identified.

A further limitation concerns incomplete procedural metadata. Aggregate participant-flow quantities and all final analytic allocations are reported where available, including the 13 Study 2 exclusions and the 19 Study 3 Time 1-to-Time 2 attrition cases. The corresponding author conducted the investigation and data curation, but the preserved de-identified research package consists of final analytic participant-level files and analysis materials rather than a complete administrative audit trail. As a result, reason-by-condition loss distributions, complete Study 1 and Study 4 screened-to-analysis flows, the original sequence-generation/assignment routine, cross-study participant-overlap status, a deterministic participant-level recommendation-mapping rule, complete practitioner-review/pretesting records, and original quality-control cutoffs cannot be recovered exactly from the retained files. These gaps are reported explicitly because reconstructing them retrospectively from final analytic data would require undocumented assumptions. They limit procedural reproducibility and the strength of any independence-based cross-study replication claim, so cross-study synthesis is presented as accumulating descriptive evidence.

Future work should manipulate or independently verify recommendation accuracy so that appropriate and inappropriate reliance can be distinguished directly, ideally while also testing rationale provision and resilience within a jointly estimated design. Field and longitudinal studies could determine whether the observed response patterns generalize beyond hypothetical scenarios and whether trust or self-efficacy trajectories predict objectively evaluated decisions over time (Pearson et al., 2026). Behavioral outcomes should also complement self-report measures. Under the present design, the most defensible conclusion is narrower: disagreement is associated with measurable differences in trust and self-efficacy, and resilience and rationale provision may alter the magnitude of those psychological responses. Whether those alterations improve calibration, performance, or welfare remains an open empirical question rather than a design recommendation.

## 6 Conclusion

Four scenario-based experiments conducted within a shared Judge-Advisor System paradigm examined how disagreement between AI-generated advice and participants’ own judgments was associated with machine trust, collaborative decision-making self-efficacy, and self-reported adaptive responses in a mixed physical-education sample. Across the reported studies, conflict was associated with lower trust and self-efficacy, with evidence consistent with indirect pathways through these measured constructs. Study 3 indicated a resilience-related attenuation of the self-efficacy association, and Study 4 showed that rationale provision moderated the condition-to-trust association, with a larger conflict-related trust reduction in the black-box condition than in the rationale-provided condition. These moderation effects should not be read as inherently protective. Without objective recommendation accuracy, the studies cannot determine whether maintaining trust reflects appropriate reliance or overreliance, nor whether maintaining self-efficacy reflects adaptive confidence or unwarranted confidence. The work is therefore best positioned as an exploratory account of psychological response patterns in human-AI disagreement rather than as evidence for algorithmic superiority, decision safety, or a validated interface intervention. Field validation, accuracy-grounded experiments, transparent materials and analysis code, and study-specific reporting remain important for evaluating generalizability and implementation.

## Data Availability

The original contributions presented in the study are included in the article/Supplementary material, further inquiries can be directed to the corresponding author.
