# Supplementary material for: When AI advice agrees or disagrees with human judgment: trust, self-efficacy, psychological resilience, and adaptive responses in physical education
Source: Front Psychol. 2026 Oct 21;17:1972538. doi: 10.3389/fpsyg.2026.1972538 (PMC13730901; doi:10.3389/fpsyg.2026.1972538)
Supplement: Supplementary file 1 [file Supplementary_file_1.docx]

**Supplementary Materials**

**Contents**

- S1. Scope and cross-study architecture
- S2. Scenario materials and experimental manipulations
- S3. Measures, response formats, and scoring
- S4. Study-specific administration and analysis details
- S5. Results crosswalk and interpretive boundaries
- S6. Data provenance, quality-control, and reproducibility notes
- S7. Selected methodological references

# S1. Scope and cross-study architecture

These Supplementary Materials accompany the four scenario-based experiments reported in the main article. The studies share a Judge-Advisor System (JAS) structure: participants first make an initial scenario judgment, then receive an experimentally presented AI recommendation that is either consistent or conflicting with that initial judgment, and subsequently complete study-specific judgments and psychological measures. The manipulation therefore concerns advice-judgment agreement rather than a direct test of AI accuracy or a data-versus-experience comparison.

The four studies form an accumulating evidence sequence rather than one pooled experiment. Study 1 tests the basic machine-trust pathway; Study 2 adds collaborative decision-making self-efficacy as a simultaneous mediator in a different scenario; Study 3 examines psychological resilience as a first-stage moderator of the condition-to-self-efficacy pathway; and Study 4 examines rationale provision as a moderator of the condition-to-machine-trust pathway. The Study 3 and Study 4 moderators were estimated in different datasets and are not combined into a single dual-moderation model.

| Study | Final analytic N | Scenario theme | Design / focal manipulation | Primary focal test |
| --- | --- | --- | --- | --- |
| Study 1 | 168 (84 consistent; 84 conflicting) | Sports injury risk alert | Single-factor between-subjects: consistent vs. conflicting | X → machine trust → adaptive response (H1–H2) |
| Study 2 | 187 (93 consistent; 94 conflicting) | Training load / tactical adjustment | Single-factor between-subjects: consistent vs. conflicting | Parallel indirect pathways through trust and self-efficacy (H3–H5) |
| Study 3 | 261 (131 consistent; 130 conflicting) | Movement technique error diagnosis | Two-wave design; resilience measured ≥48 h before JAS experiment | Resilience moderates X → self-efficacy and its indirect pathway (H6–H7) |
| Study 4 | 243 (61 C/BB; 61 X/BB; 61 C/R; 60 X/R) | Integrated training decision | 2 × 2: consistency × rationale provision | Rationale provision moderates X → machine trust (H8a only) |

Eligibility and population. Participants were drawn from a mixed physical-education population and were required to have at least one year of teaching or coaching experience. Eligible groups included K–12 physical-education teachers, university-level physical-education instructors, sport-specific coaches, and senior undergraduate or graduate physical-education majors with at least one cumulative year of internship or part-time coaching experience. Population-level interpretation is therefore limited to this mixed eligible sample rather than experienced in-service teachers alone.

Analytic scope. All focal results refer to the retained complete-case/per-protocol samples. The article does not claim preregistration, does not pool p values across studies, does not apply multiplicity adjustment, and does not treat the exploratory AI-adoption measure as evidence for H1–H8a.

# S2. Scenario materials and experimental manipulations

The proof states that the original Supplementary Materials contain the full scenario texts in the original language of administration and translated versions. The exact archived wording is not present in the proof. The scenario texts below are therefore publication-structure drafts that reproduce the scenario themes, JAS sequence, manipulation logic, and inferential boundaries stated in the proof. They must be checked against the archived materials before submission.

## **S2.1 Common JAS sequence**

1. Participants read a physical-education or coaching scenario and rendered an initial numerical judgment.
2. An AI recommendation was then presented. The recommendation was experimentally assigned to be consistent or conflicting relative to the participant’s immediately stated judgment.
3. Pretesting targeted an approximately two-point discrepancy for the conflicting condition as a calibration goal. The main article explicitly does not claim that every participant received a fixed ±2-point arithmetic offset, and the preserved records do not support a deterministic participant-level yoking or endpoint rule.
4. Participants then rendered a final judgment. Directional Weight of Advice (WOA), where used, was defined as (final judgment − initial judgment)/(AI recommendation − initial judgment), contingent on a nonzero denominator.
5. Post-manipulation measures were administered according to the study-specific measurement matrix below. The AI feedback was an experimental manipulation rather than genuine system output, and participants were debriefed after participation.

### **S2.2 Study 1 — Sports injury risk alert**

**English draft reconstruction**

Scenario. You are responsible for a physical-education session or training activity. A participant/student has recently completed a demanding activity and shows several observable indicators that could be relevant to short-term injury risk. You must decide how concerned you are about the risk associated with continuing the planned activity.

Initial judgment prompt. Based on the information available in the scenario, indicate your own assessment of the participant’s current injury risk using the numerical response scale provided in the study.

AI recommendation. After the initial judgment, an AI-based decision-support system presents its own risk recommendation. In the consistent condition, the AI recommendation is directionally aligned with the participant’s initial judgment. In the conflicting condition, the recommendation is calibrated to be meaningfully discrepant from the participant’s initial judgment, with pretesting targeting approximately a two-point discrepancy as a general calibration goal rather than a fixed rule.

Final judgment prompt. After viewing the AI recommendation, indicate your final judgment. Participants then completed the post-manipulation machine-trust and collaborative adaptive-response measures used in Study 1.

**Draft Chinese administration-language version**

情境。你正在负责一节体育课或训练活动。一名学生/参与者刚完成强度较高的活动，并出现若干可能与短期运动损伤风险有关的可观察信息。你需要判断继续按原计划活动时，其当前损伤风险值得多大程度的关注。

初始判断。请仅根据当前情境信息，在研究所使用的数值量表上给出你本人对该学生/参与者当前损伤风险的判断。

AI 建议。在你完成初始判断后，一个 AI 决策支持系统会给出其风险建议。在“一致”条件下，AI 建议与参与者的初始判断方向一致；在“冲突”条件下，AI 建议与参与者的初始判断存在有意义的差异。预试阶段以约两个量表点的差异作为总体校准目标，但并非对每名参与者固定使用 ±2 的算术偏移。

最终判断。在查看 AI 建议后，请再次给出你的最终判断。随后完成 Study 1 所使用的机器信任与协作适应反应测量。

### **S2.3 Study 2 — Training load / tactical adjustment**

**English draft reconstruction**

Scenario. You are planning a physical-education or sport-training session in which the current training load and/or tactical plan may need adjustment. Available observations provide plausible reasons both for maintaining the original plan and for modifying it.

Initial judgment prompt. Indicate your own judgment about the appropriate degree of training-load or tactical adjustment using the numerical response scale provided in the study.

AI recommendation. The AI-based decision-support system then presents a recommendation that is either consistent or conflicting with the participant’s initial judgment. The conflicting recommendation is calibrated to create a salient but plausible discrepancy, following the same general calibration approach used in Study 1.

Final judgment and post-manipulation measures. Participants provide a final judgment after seeing the recommendation. Machine trust and collaborative decision-making self-efficacy are then assessed in randomized presentation order, followed by the collaborative adaptive-response measure.

**Draft Chinese administration-language version**

情境。你正在安排一节体育课或专项训练，本次训练的负荷和/或战术安排可能需要调整。当前信息既存在维持原计划的合理依据，也存在进行调整的合理依据。

初始判断。请在研究所使用的数值量表上，给出你本人认为训练负荷或战术安排应当调整到何种程度。

AI 建议。随后，AI 决策支持系统给出一条建议。该建议根据实验条件被设置为与参与者的初始判断“一致”或“冲突”。冲突条件采用与 Study 1 相同的总体校准原则，使差异足够明显但仍保持情境上的合理性。

最终判断与后续测量。参与者查看 AI 建议后给出最终判断。随后以随机顺序呈现机器信任与人机协作决策自我效能量表，最后测量协作适应反应。

### **S2.4 Study 3 — Movement technique error diagnosis**

**English draft reconstruction**

Time 1. At least 48 hours before the experimental session, participants completed the psychological-resilience measure and demographic items.

Time 2 scenario. You are evaluating a learner or athlete performing a movement skill. The observed performance contains cues that could be interpreted as a technique error requiring corrective action, but the available information does not establish an objectively verified ground truth.

Initial judgment prompt. Indicate your own judgment regarding the severity or corrective importance of the observed movement-technique problem using the numerical response scale provided in the study.

AI recommendation and final judgment. The AI system provides a recommendation assigned to be consistent or conflicting with the participant’s initial judgment under the same general calibration principle as the earlier studies. After viewing the recommendation, participants provide a final judgment and complete post-manipulation machine trust, collaborative decision-making self-efficacy, and collaborative adaptive-response measures. Trust and self-efficacy are presented in randomized order.

**Draft Chinese administration-language version**

时间点 1。在正式实验至少 48 小时之前，参与者完成心理韧性量表与人口统计学信息。

时间点 2 情境。你正在评估一名学生或运动员完成某项动作技能的表现。观察到的信息可能提示存在需要纠正的技术错误，但当前情境并未提供一个客观验证的“唯一正确答案”。

初始判断。请在研究所使用的数值量表上，给出你本人对该动作技术问题严重程度或纠正必要性的判断。

AI 建议与最终判断。AI 系统按照与前述研究相同的总体校准原则，给出与参与者初始判断一致或冲突的建议。查看建议后，参与者完成最终判断，并完成机器信任、人机协作决策自我效能和协作适应反应测量；机器信任与自我效能的呈现顺序随机化。

### **S2.5 Study 4 — Integrated training decision and rationale provision**

**English draft reconstruction**

Scenario. You are making an integrated physical-education or training decision that requires balancing several pieces of information, such as recent performance, workload, observable condition, and the immediate instructional or training objective.

Initial judgment prompt. Indicate your own preferred decision or adjustment on the numerical response scale used in the study.

Consistency manipulation. The AI system provides a judgment outcome that is either consistent or conflicting with the participant’s initial judgment.

Rationale manipulation. In the black-box condition, participants receive the AI judgment outcome without an accompanying explanation. In the rationale-provided condition, the same type of AI judgment outcome is accompanied by a concise explanation referring to the scenario information considered by the system. All four cells receive an AI judgment outcome; only the two rationale-provided cells receive explanatory information.

Post-manipulation outcome. Machine trust is assessed after the 2 × 2 manipulation. Study 4 does not include a pre-manipulation trust measure, so the reported differences are between-condition means rather than within-person trust changes.

**Draft Chinese administration-language version**

情境。你需要做出一个综合性的体育教学或训练决策，需要同时权衡近期表现、训练负荷、可观察的身体/动作状态以及当前教学或训练目标等多项信息。

初始判断。请在研究所使用的数值量表上给出你本人倾向采用的决策或调整程度。

一致性操纵。AI 系统给出一个判断结果，该结果根据实验条件被设置为与参与者初始判断一致或冲突。

理由操纵。在“黑箱”条件下，参与者只看到 AI 的判断结果，不提供解释；在“提供理由”条件下，参与者看到同类型的 AI 判断结果，同时获得一段简短解释，说明系统在该情境下考虑了哪些信息。四个实验单元均提供 AI 判断结果，只有“提供理由”的两个单元额外提供解释信息。

后续测量。完成 2 × 2 操纵后测量机器信任。Study 4 没有操纵前机器信任测量，因此正文报告的是条件间均值差异，而不是个体内的信任变化。

## **S2.6 Manipulation-check wording**

The main article reports an agreement manipulation check in which participants in the conflicting condition rated the agreement between the AI judgment and their initial scenario judgment lower than participants in the consistent condition. A publication-ready supplement should reproduce the exact check item and response anchors used in the study.

# S3. Measures, response formats, and scoring

The proof identifies four focal measures: a six-item context-adapted machine-trust scale, a seven-item collaborative decision-making self-efficacy scale, the 10-item Chinese CD-RISC psychological-resilience measure, and a five-item collaborative adaptive-response scale. It also discloses a four-item exploratory AI-adoption-intention measure whose study-specific administration timing is not documented. The exact item wording, anchors, and scoring rules are not reproduced in the proof. The sections below therefore separate source-grounded construct definitions from reconstructed draft wording.

## **S3.1 Machine trust (6 items)**

Source basis. Context-adapted from the human-automation trust scale of Jian, Bisantz, and Drury (2000). In the main article, machine trust is interpreted as a psychological judgment about the AI system’s competence, reliability, and dependability; it is not equated with appropriate reliance or decision accuracy.

6. MT1. I consider the AI recommendation in this scenario dependable. [RECONSTRUCTED — AUTHOR VERIFY]
7. MT2. I feel that I can rely on the AI system when evaluating this scenario. [RECONSTRUCTED — AUTHOR VERIFY]
8. MT3. The AI recommendation appears competent for supporting this decision. [RECONSTRUCTED — AUTHOR VERIFY]
9. MT4. I trust the AI system to provide useful decision support in this situation. [RECONSTRUCTED — AUTHOR VERIFY]
10. MT5. The AI recommendation seems reliable enough to take into account. [RECONSTRUCTED — AUTHOR VERIFY]
11. MT6. Overall, I have confidence in the AI system’s recommendation in this scenario. [RECONSTRUCTED — AUTHOR VERIFY]

## **S3.2 Collaborative decision-making self-efficacy (7 items)**

Construct basis. Grounded in Bandura’s self-efficacy framework and contextualized to evaluating discrepant information and reaching a workable decision with AI support. The construct concerns perceived capability rather than objective decision performance.

12. SE1. I am confident that I can evaluate my own judgment together with the AI recommendation. [RECONSTRUCTED — AUTHOR VERIFY]
13. SE2. I can identify the important points of agreement or disagreement between my judgment and the AI advice. [RECONSTRUCTED — AUTHOR VERIFY]
14. SE3. I can decide how much weight to give the AI recommendation when it differs from my initial view. [RECONSTRUCTED — AUTHOR VERIFY]
15. SE4. I can integrate relevant information from the AI recommendation without losing sight of the situational context. [RECONSTRUCTED — AUTHOR VERIFY]
16. SE5. I can reach a workable final decision even when the AI recommendation conflicts with my initial judgment. [RECONSTRUCTED — AUTHOR VERIFY]
17. SE6. I can explain the reasoning behind my final decision after considering the AI recommendation. [RECONSTRUCTED — AUTHOR VERIFY]
18. SE7. I can manage uncertainty effectively when making a decision together with AI support. [RECONSTRUCTED — AUTHOR VERIFY]

## **S3.3 Psychological resilience (Chinese CD-RISC-10)**

Source basis. The main article states that psychological resilience was assessed with the 10-item Chinese CD-RISC, citing Connor and Davidson (2003) and Campbell-Sills and Stein (2007). In Study 3 it was measured at Time 1, at least 48 hours before the JAS experiment, and treated as a premeasured individual-difference resource rather than an immutable trait or an observed recovery process.

| Item | Exact administered wording |
| --- | --- |
| R1 | [AUTHOR VERIFY — insert exact authorized Chinese CD-RISC-10 item] |
| R2 | [AUTHOR VERIFY — insert exact authorized Chinese CD-RISC-10 item] |
| R3 | [AUTHOR VERIFY — insert exact authorized Chinese CD-RISC-10 item] |
| R4 | [AUTHOR VERIFY — insert exact authorized Chinese CD-RISC-10 item] |
| R5 | [AUTHOR VERIFY — insert exact authorized Chinese CD-RISC-10 item] |
| R6 | [AUTHOR VERIFY — insert exact authorized Chinese CD-RISC-10 item] |
| R7 | [AUTHOR VERIFY — insert exact authorized Chinese CD-RISC-10 item] |
| R8 | [AUTHOR VERIFY — insert exact authorized Chinese CD-RISC-10 item] |
| R9 | [AUTHOR VERIFY — insert exact authorized Chinese CD-RISC-10 item] |
| R10 | [AUTHOR VERIFY — insert exact authorized Chinese CD-RISC-10 item] |

## **S3.4 Collaborative adaptive response (5 items)**

Construct role. This five-item self-report scale is the primary outcome used to represent participants’ intended collaborative adaptive responses after considering the AI recommendation. The main article intentionally avoids treating this measure as objective decision quality, welfare, or safety.

19. AR1. I would reconsider my initial judgment in light of the information provided by the AI. [RECONSTRUCTED — AUTHOR VERIFY]
20. AR2. I would adjust my decision process when the AI recommendation highlights information I may have underweighted. [RECONSTRUCTED — AUTHOR VERIFY]
21. AR3. I would actively compare the AI recommendation with my own situational judgment before deciding what to do. [RECONSTRUCTED — AUTHOR VERIFY]
22. AR4. I would be willing to revise my planned action when the combined information supports an adjustment. [RECONSTRUCTED — AUTHOR VERIFY]
23. AR5. I would continue to use both professional judgment and AI input flexibly when responding to this situation. [RECONSTRUCTED — AUTHOR VERIFY]

## **S3.5 Directional Weight of Advice (WOA)**

WOA = (final judgment − initial judgment) / (AI recommendation − initial judgment). In the article, WOA is treated as a descriptive advice-taking indicator rather than objective decision quality. Its interpretability depends on a nonzero advice-minus-initial-judgment denominator.

## **S3.6 Exploratory AI adoption intention (4 items)**

A four-item AI-adoption-intention measure formed part of the exploratory project materials. The retained study materials do not document its study-specific administration timing, so the main article does not assign it to a particular study and does not use it in focal psychometric, mediation, moderation, or causal claims.

24. AI1. [AUTHOR VERIFY — insert exact exploratory AI adoption-intention item]
25. AI2. [AUTHOR VERIFY — insert exact exploratory AI adoption-intention item]
26. AI3. [AUTHOR VERIFY — insert exact exploratory AI adoption-intention item]
27. AI4. [AUTHOR VERIFY — insert exact exploratory AI adoption-intention item]

# S4. Study-specific administration and analysis details

| Study / time point | Variables explicitly reported | Administration / analysis sequence |
| --- | --- | --- |
| Study 1 — single session | Initial judgment; AI recommendation; final judgment/WOA; machine trust; collaborative adaptive response | Initial judgment → AI recommendation → final judgment. Trust and adaptive-response measures followed the manipulation. Independent-samples tests and PROCESS Model 4 with 5,000 bootstrap resamples. |
| Study 2 — single session | Initial judgment; AI recommendation; final judgment/WOA; machine trust; collaborative decision-making self-efficacy; adaptive response | JAS sequence as above. Trust and self-efficacy presentation order randomized; adaptive response assessed post-manipulation. Parallel PROCESS Model 4 with 5,000 bootstrap resamples. |
| Study 3 — Time 1 | CD-RISC-10 resilience; demographics | Administered at least 48 h before Time 2. |
| Study 3 — Time 2 | JAS sequence; machine trust; self-efficacy; adaptive response | Trust/self-efficacy order randomized. First-stage conditional-process model; self-efficacy equation included condition, resilience, and interaction; adaptive-response equation included condition, self-efficacy, and machine trust. 5,000 bootstrap resamples. |
| Study 4 — single session | JAS sequence; rationale-provision manipulation; machine trust | 2 × 2 condition × rationale-provision procedure; factorial OLS ANOVA and simple-effects comparisons. H8a only; no moderated mediation through trust is claimed. |
| Exploratory project measure | AI adoption intention (4-item scale) | Study-specific timing not documented in retained materials; excluded from focal hypothesis testing. |

## **S4.1 Study 4 factorial-cell description**

| Consistency | Rationale provision | Final n | Machine trust M (SD) |
| --- | --- | --- | --- |
| Consistent | Black-box | 61 | 5.260 (0.69) |
| Conflicting | Black-box | 61 | 4.448 (0.72) |
| Consistent | Rationale provided | 61 | 5.238 (0.76) |
| Conflicting | Rationale provided | 60 | 5.056 (0.72) |

The reported conflict effect was −0.811 in the black-box condition and −0.182 in the rationale-provided condition. Because no pre-manipulation trust measure was administered, these are between-condition contrasts rather than within-person change scores.

# S5. Results crosswalk and interpretive boundaries

This section is included to ensure that the supplementary materials map cleanly onto the main-text methods and conclusions. It does not introduce new analyses or expand the inferential scope of the article.

| Claim / hypothesis | Study | Reported result | Interpretive boundary retained in supplement |
| --- | --- | --- | --- |
| H1: conflict associated with lower machine trust | Study 1 | t(166)=6.87, p<0.001, d=1.061 | Lower trust is not automatically treated as maladaptive or as algorithm aversion. |
| H2: indirect pathway through trust | Study 1 | B=−0.428, Boot SE=0.079, 95% CI [−0.591, −0.286] | Measured mediator; consistent with mediation, not definitive proof of a causal psychological mechanism. |
| H3: conflict associated with lower self-efficacy | Study 2 | B=−0.687, SE=0.132, p<0.001 | Lower self-efficacy may reflect warranted uncertainty as well as disruptive self-doubt. |
| H4/H5: simultaneous indirect pathways | Study 2 | Trust B=−0.201, 95% CI [−0.296, −0.118]; self-efficacy B=−0.127, 95% CI [−0.207, −0.058] | Parallel specification does not establish causal or statistical independence between mediators. Contrast CI crossed zero. |
| H6/H7: resilience attenuation | Study 3 | Condition×resilience B=0.276, p=0.001; moderated-mediation index=0.115, 95% CI [0.041, 0.203] | Between-condition buffering pattern, not within-person recovery. No claim that maintained confidence improves accuracy. |
| H8a: rationale provision moderates condition→trust | Study 4 | F(1,239)=11.348, p<0.001, partial η²=0.045 | Effect is provision of a rationale, not explainability in general. No outcome-level moderated mediation is claimed. |

## **S5.1 Cross-study synthesis**

Across the four studies, the reported coefficients are directionally consistent after harmonizing condition coding (0 = consistent, 1 = conflicting). Study 3 provides evidence that resilience moderates the self-efficacy pathway, whereas Study 4 shows that rationale provision moderates the trust pathway. These findings are accumulating, study-specific evidence; they are not one jointly estimated dual-moderation model and are not presented as an independence-based replication claim.

The central interpretive boundary is preserved throughout the supplement: recommendation accuracy was not manipulated or objectively established. Accordingly, smaller reductions in trust or self-efficacy cannot be labeled inherently beneficial, and higher trust, higher reliance, or higher confidence cannot be assumed to indicate better calibration, decision quality, safety, or welfare.

# S6. Data provenance, quality-control, and reproducibility notes

- The preserved research files are de-identified final analytic participant-level datasets rather than complete platform-level administrative audit trails.
- Complete pre-analysis screening histories for Studies 1 and 4 are not reconstructible from the retained analytic files.
- Study 2 reports 13 aggregate data-quality exclusions; the reason-by-condition distribution and historical numerical thresholds are not preserved.
- Study 3 reports 19 Time 1-to-Time 2 attrition cases; a reason-by-condition attrition account is not preserved.
- The original sequence-generation or allocation log is not retained; the article therefore reports observed allocations without claiming simple, blocked, stratified, or another unverified randomization mechanism.
- The original deterministic participant-level recommendation mapping or endpoint rule is not preserved. The conflict manipulation is therefore reported categorically, with approximately two points described only as a pretesting calibration goal.
- Practitioner review is treated as a qualitative face-validity check. A complete reviewer roster, standardized rating instrument, inter-rater statistic, and participant-level pretesting flow are not available in the retained records.
- The studies are not presented as preregistered. No multiplicity-adjustment procedure is claimed; p values are interpreted together with effect sizes and confidence intervals.
- Harman’s single-factor test is treated only as a descriptive diagnostic and is not used to claim that common-method variance was absent.
- Primary analyses are presented without covariate adjustment; candidate covariates were recorded but are not used to redefine the focal results.

## **S6.1 Participant flow**

| Study | Flow information supported by retained records | Final analytic allocation |
| --- | --- | --- |
| Study 1 | Pre-analysis screened-to-analysis flow not reconstructible | 168; 84 consistent, 84 conflicting |
| Study 2 | 200 entered study flow; 13 data-quality exclusions | 187; 93 consistent, 94 conflicting |
| Study 3 | 280 completed Time 1; 19 lost before Time 2 | 261; 131 consistent, 130 conflicting |
| Study 4 | Pre-analysis screened-to-analysis flow not reconstructible | 243; 61 C/BB, 61 X/BB, 61 C/R, 60 X/R |

# S7. Selected methodological references

Bandura, A. (2006). “Guide for constructing self-efficacy scales,” in Self-Efficacy Beliefs of Adolescents, eds. F. Pajares and T. Urdan (Greenwich: Information Age Publishing).

Bonaccio, S., and Dalal, R. S. (2006). Advice taking and decision-making: an integrative literature review, and implications for the organizational sciences. Organizational Behavior and Human Decision Processes, 101, 127–151. doi: 10.1016/j.obhdp.2006.07.001.

Campbell-Sills, L., and Stein, M. B. (2007). Psychometric analysis and refinement of the Connor-Davidson resilience scale (CD-RISC): validation of a 10-item measure of resilience. Journal of Traumatic Stress, 20, 1019–1028. doi: 10.1002/jts.20271.

Collie, R. J., Martin, A. J., and Gašević, D. (2024). Teachers’ generative AI self-efficacy, valuing, and integration at work: examining job resources and demands. Computers and Education: Artificial Intelligence, 7, 100333. doi: 10.1016/j.caeai.2024.100333.

Connor, K. M., and Davidson, J. R. T. (2003). Development of a new resilience scale: the Connor-Davidson resilience scale (CD-RISC). Depression and Anxiety, 18, 76–82. doi: 10.1002/da.10113.

Harvey, N., and Fischer, I. (1997). Taking advice: accepting help, improving judgment, and sharing responsibility. Organizational Behavior and Human Decision Processes, 70, 117–133. doi: 10.1006/obhd.1997.2697.

Hayes, A. F. (2022). Introduction to Mediation, Moderation, and Conditional Process Analysis: A Regression-Based Approach. New York: Guilford Press.

Jian, J. Y., Bisantz, A. M., and Drury, C. G. (2000). Foundations for an empirically determined scale of trust in automated systems. International Journal of Cognitive Ergonomics, 4, 53–71. doi: 10.1207/S15327566IJCE0401_04.
